# Tunable Band Gap and Conductivity Type of ZnSe/Si Core-Shell Nanowire Heterostructures

**DOI:** 10.3390/ma7117276

**Published:** 2014-10-31

**Authors:** Yijie Zeng, Huaizhong Xing, Yanbian Fang, Yan Huang, Aijiang Lu, Xiaoshuang Chen

**Affiliations:** 1 Department of Applied Physics and State Key Laboratory for Modification of Chemical Fibers and Polymer Materials, Donghua University, Ren Min Road 2999, Songjiang District, Shanghai 201620, China; E-Mails: dhu_zyj@mail.dhu.edu.cn (Y.Z.); fangyanbian@163.com (Y.F.); ajlu@dhu.edu.cn (A.L.); 2 National Lab of Infrared Physics, Shanghai Institute for Technical Physics, Chinese Academy of Science, 500 Yu Tian Road, Shanghai 200083, China; E-Mail: yhuang@mail.sitp.ac.cn

**Keywords:** ZnSe/Si core-shell, nanowires, interface states, tunable bandgap, conductivity type

## Abstract

The electronic properties of zincblende ZnSe/Si core-shell nanowires (NWs) with a diameter of 1.1–2.8 nm are calculated by means of the first principle calculation. Band gaps of both ZnSe-core/Si-shell and Si-core/ZnSe-shell NWs are much smaller than those of pure ZnSe or Si NWs. Band alignment analysis reveals that the small band gaps of ZnSe/Si core-shell NWs are caused by the interface state. Fixing the ZnSe core size and enlarging the Si shell would turn the NWs from intrinsic to *p*-type, then to metallic. However, Fixing the Si core and enlarging the ZnSe shell would not change the band gap significantly. The partial charge distribution diagram shows that the conduction band maximum (CBM) is confined in Si, while the valence band maximum (VBM) is mainly distributed around the interface. Our findings also show that the band gap and conductivity type of ZnSe/Si core-shell NWs can be tuned by the concentration and diameter of the core-shell material, respectively.

## 1. Introduction

Semiconductor nanowires (NWs) have received extensive attention for their unique one-dimensional structure and the progress in the preparation method [1]. The past decade has witnessed their application in various electronic and photonic devices, such as diodes [2], lasers [3], field effect transistors [4], biological sensors [5] and integrated logical calculators [6,7]. Among all kinds of NWs, core-shell NWs are prominent for their variation of composition in the radial direction [8]. Since NWs are well known for their ability to form heterostructures with large lattice mismatch, while avoiding creating dislocations, core-shell NWs are hoped to be the carrier of heterostructures that cannot be grown epitaxially in planar form [9].

Silicon (Si) is the basic material in the semiconductor industry, and mature methods have been established to fabricate Si-based electronics. Zinc selenide (ZnSe) is widely used in photocatalytic activities [10] and short-wavelength optoelectronics [11,12] for its large bandgap (E_g_ = 2.67 eV at 300 K) and excitonic binding energy [13]. ZnSe NWs have been prepared by the vapor-liquid-solid method [14,15] and the solution-based method [16,17]. It is well known that the shape and diameter are two features for tuning the electronic properties of one-dimensional nanostructures. However, if the composition varies along the radial direction, namely the core and shell parts of NWs are composed of different materials, new phenomena would occur, due to the band alignment and strain caused by lattice mismatch on the interface. To see their effects on the electronic properties of NWs, some attempts have been made: for example, coaxial ZnSe/Si nanocables with high tunability of the shell conductivity have been synthesized by varying the boron concentration in the shell [18], and silica-sheathed ZnSe nanostructures are found to have improved stability and to not have their optical properties influenced [19].

Previous theoretical calculations focused on ZnSe/Si coaxial NWs [20] or pure Si NWs [21,22,23], while few talked about ZnSe/Si core-shell NWs. Therefore, in this paper, we present the electronic properties of ZnSe NWs, nanotubes (NTs) and ZnSe/Si core-shell NWs by means of the first principles calculation based on density functional theory (DFT) [24,25]. The results show that ZnSe NTs have larger band gaps than those of ZnSe NWs of the same diameter, due to their stronger quantum confinement effect. Both ZnSe NWs and Si NWs have larger band gaps than their bulk counterparts. However, both ZnSe-core/Si-shell and Si-core/ZnSe-shell NWs have much smaller band gaps than those of ZnSe and Si NWs with the same diameter. Further analysis of the partial charge densities reveals that ZnSe/Si core-shell NWs have type I band alignment and interface states caused by the large lattice mismatch between ZnSe and Si. 

## 2. Theoretical Method 

The DFT calculations are performed with Vienna Ab initio Simulation Package (VASP) code [26], using the generalized gradient approximation (GGA) suggested by Perdew, Burke and Ernzerhof (PBE) [27]. The wave functions are expanded on a plane wave basis with kinetic energy cutoff set to 360 eV. A Monkhorst–Pack scheme [28] *k* point mesh of 1 × 1 × 6 is used for the first Brillouin zone integration. All of the structures are fully optimized until the allowed error in the total energy is less than 10^−4^ eV and the error in the forces is smaller than 2 × 10^−2^ eV/Å. To eliminate the interactions between neighboring NWs, a supercell of a 10~15 Å separation distance is applied to the structures. During the optimization, no symmetry constraints are used.

**Figure 1:**
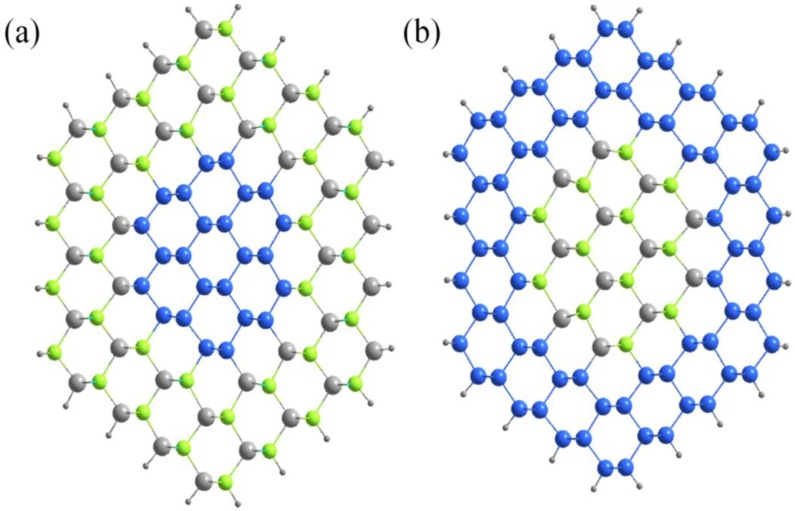
Top view of a typical (**a**) Si-core/ZnSe-shell and (**b**) ZnSe-core/Si-shell NWs along the [110] direction. The blue, grey and green spheres represent Si, Zn and Se atoms, respectively. The outer black spheres are pseudo hydrogen atoms.

**Figure 2:**
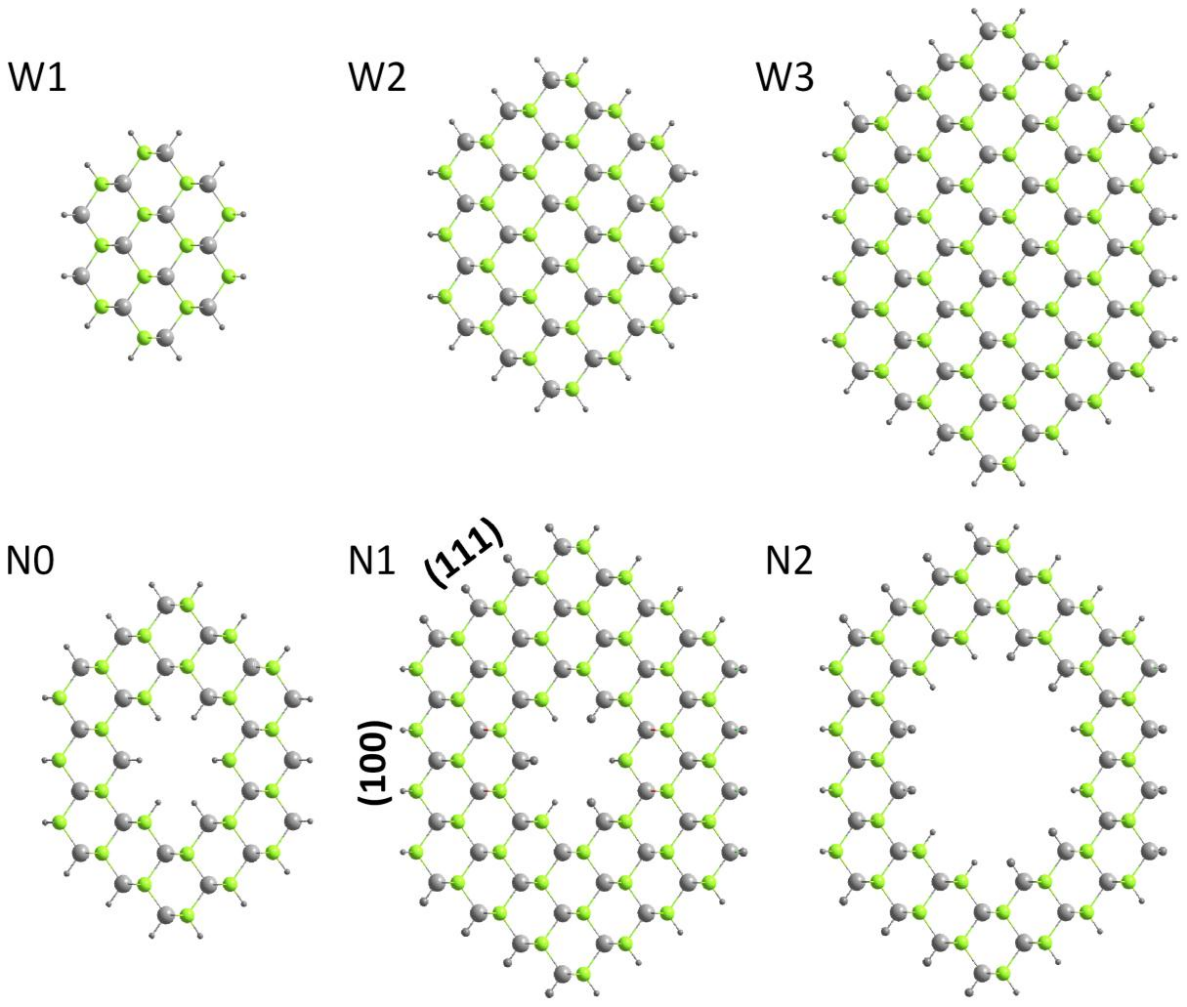
Top view of ZnSe nanowires (W1–W3) and nanotubes (N0–N2). The grey and green spheres represent Zn and Se atoms, while the outer and inner black spheres are pseudo hydrogen atoms.

The details about the construction of the model of ZnSe-core/Si-shell NWs are described in our former works [29]. In short, only zincblende ZnSe is considered, and the direction is along [110] for the experimental findings [30]. ZnSe-core/Si-shell NWs with diameter ranges of 1.1–2.8 nm are considered, and they have hexagonal shapes, with the surface atoms saturated by hydrogen atoms. Si-core/ZnSe-shell NWs are the same, apart from the inner ZnSe being replaced by Si, and the surface Zn and Se atoms are saturated by pseudo hydrogen atoms with a fractional charge of 1.5 *e* and 0.5 *e*, respectively. Then, atoms in the unit cell can be classified into the core and shell part; thus, we define the total number of atoms per unit cell as *N* = *N*_core_ + *N*_shell_, where hydrogen atoms are not considered. In this way, an NW can be identified uniquely by ZnSe-core (*R*, *x*) or Si-core (*R*, *x*), where *R* is the diameter and *x* = *N*_core_/(*N*_core_ + *N*_shell_). Representative schemes of ZnSe-core/Si-shell and Si-core/ZnSe-shell NWs are shown in Figure 1. ZnSe NTs are built from ZnSe NWs by removing the inner Zn and Se atoms and identified as ZnSe-NT (*R*, *x*), where *x* is the ratio of missing atoms. All of the considered NTs have at least two Zn-Se bilayers, and all of the surface atoms have been saturated by pseudo hydrogen atoms. Figure 2 shows the top view of the ZnSe NWs and NTs considered.

## 3. Results and Discussion

**Figure 3:**
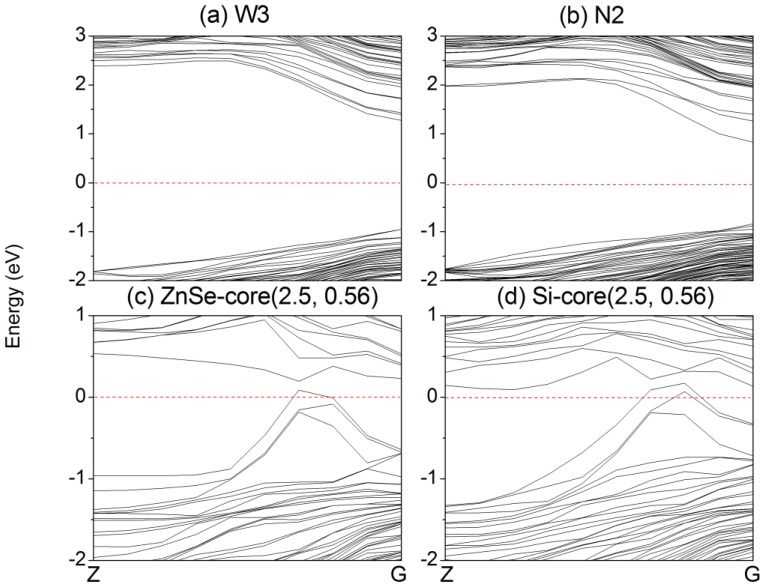
Band structure of ZnSe (**a**) NWs W3; (**b**) NTs N2 and (**c**) ZnSe-core (2.5, 0.56) NW; (**d**) Si-core (2.5, 0.56) NW. The red horizontal dashed line is the Fermi energy, which is set to zero.

**Figure 4:**
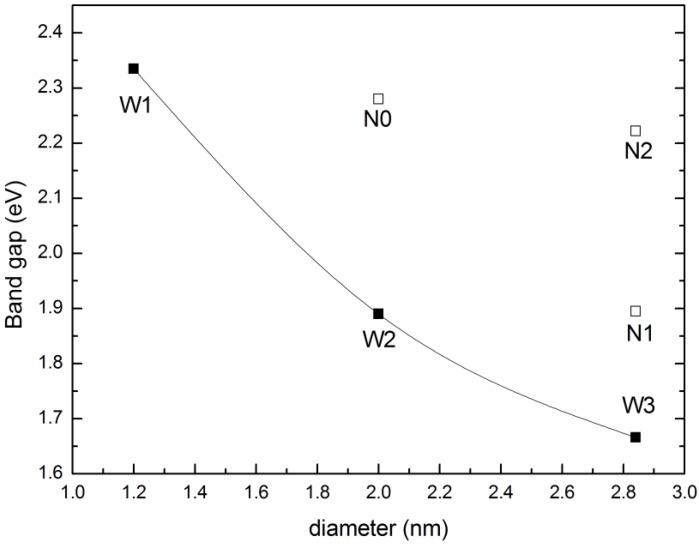
Variation of the band gaps of ZnSe NWs and NTs with the diameter; terms in the figure correspond to the structures shown in Figure 2.

Firstly, the electronic properties of ZnSe NWs and NTs are analyzed. The band structures of representative ZnSe NWs (W3) and NTs (N2) are displayed in Figure 3a,b. The band gap of bulk ZnSe is 1.356 eV, which is smaller than the experimental value (2.70 eV) [31]. This can be anticipated, since DFT calculations underestimate the value of band gaps. However, the trend of the band gaps calculated under the same accuracy is valid. Figure 4 gives the variation trend of the band gaps of NWs and NTs. All of the calculated NWs have direct band gaps, and the band gap decreases as the diameter of the NW increases and, finally, approaches the bulk value. For example, the band gap of ZnSe NWs with a diameter of 2 nm is 1.89 eV and decreases to 1.67 eV when the diameter is 2.84 nm. This is caused by the quantum confinement effect [32]. While for ZnSe NTs, the inner loop offers another degree of size tuning. When the diameter of the outer loop is unchanged, the band gap of NTs increases with increasing of the diameter of inner loop, such as the variation from N1 to N2. Since the cross-section area of NTs is smaller than that of NWs with the same diameter, the band gap of NTs would be larger than that of NWs at a given diameter, due to the enhanced quantum confinement effect, as confirmed by, for example, W3 and N2. A similar trend has also been observed in Si NWs and NTs.

**Figure 5:**
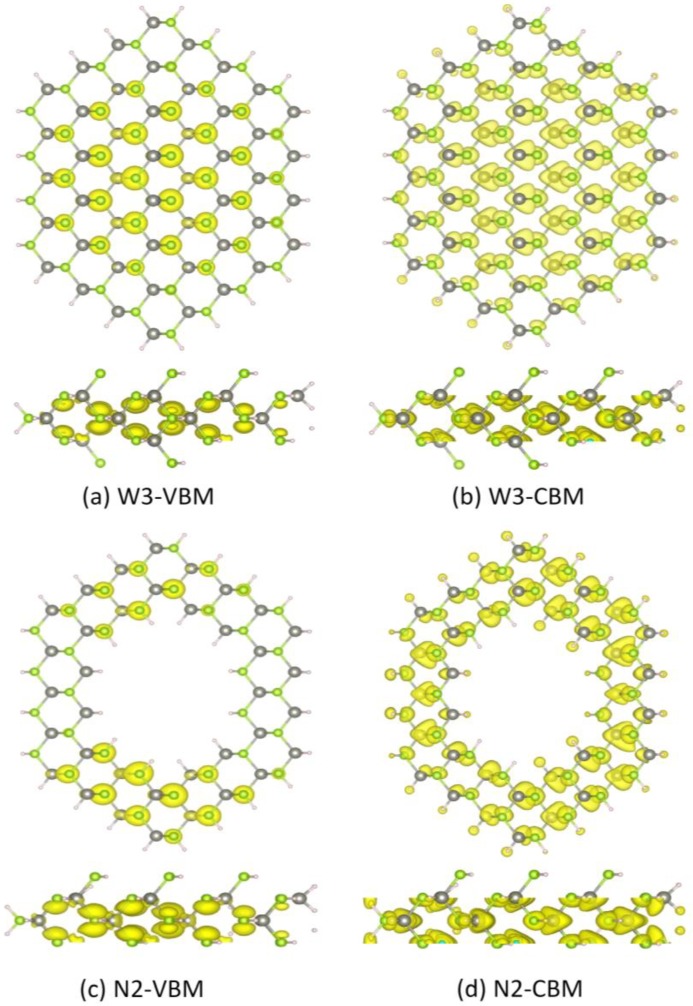
Partial charge density of the valence band maximum (VBM) and conduction band minimum (CBM) of ZnSe NWs (W3) and NTs (N2) at the G point. (**a**) W3-VBM; (**b**) W3-CBM; (**c**) N2-VBM; (**d**) N2-CBM.

Figure 5 shows the partial charge density distribution of the valence band maximum (VBM) and conduction band minimum (CBM) of typical ZnSe NWs (W3) and NTs (N2). The VBM of W3 mainly comes from the 4*p* orbital of the inner Se atoms, while the CBM primarily originates from the 4*s* orbital of the inner Se atoms and the *sp^3^* orbital, consisting of the Zn 4*s* and Se 4*p* orbitals. For NT2, the CBM also originates from the 4*s* orbital of the Se atoms and the *sp^3^* orbital. However, the VBM mainly comes from the 4*p* orbital of the Se atoms that lie on the (111) and (211) facets. This can be explained by the fact that the VBM of N2 is degenerate, while the VBM of W3 is not, which can be seen in Figure 3.

**Figure 6:**
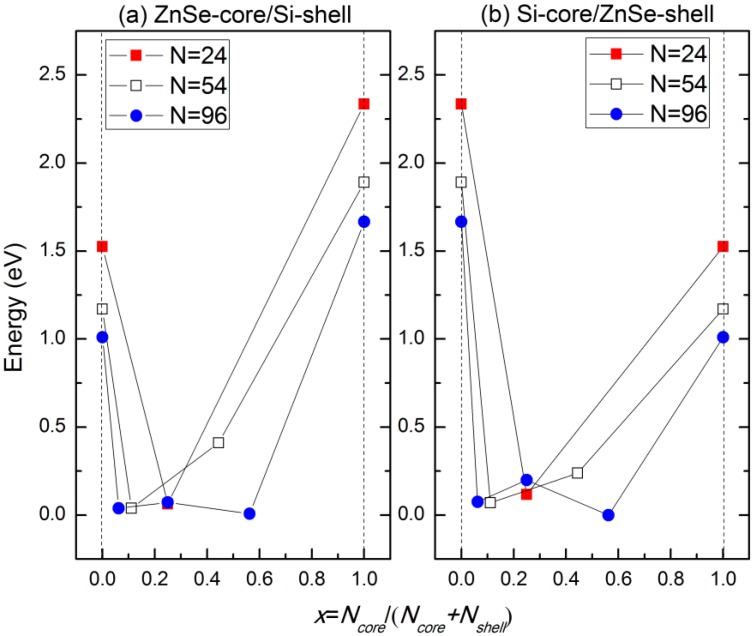
Variation of the band gaps of (**a**) ZnSe-core/Si-shell and (**b**) Si-core/ZnSe-shell NWs with the ratio of core atoms.

The variation of the band gaps of the ZnSe-core/Si-shell NWs and Si-core/ZnSe-shell NWs with the diameter and ratio of the core atoms is displayed in Figure 6. It can be seen that when radial heterostructures are formed, the band gap would be smaller than that of the Si and ZnSe NWs of the same diameter, as indicated by the solid line in both Figure 6a,b. This is contrary to the derivational band alignment of the bulk phases of ZnSe and Si, where they would form the type I heterostructure, namely Si has a higher VBM and a lower CBM simultaneously, and the band gap of the ZnSe/Si core-shell NWs would be close to that of Si. We can eliminate the influence of surface dangling bonds, which have been saturated by pseudo hydrogen atoms. Besides, some structures of the ZnSe-core/Si-shell NWs, for example the ZnSe-core (2.5, 0.06~0.6), show *p*-type characteristics, namely the VBM crosses the Fermi energy level, as indicated in Figure 3c. Moreover, the Si-core (2.5, 0.56) NW becomes metallic, as shown in Figure 3d. We further increase the diameter and find that the NWs would become metallic for both the ZnSe-core and Si-core NWs. This may be the reason why it is hard to get *p*-type NWS in the experiment. In addition, this means that by varying the concentration of core atoms and the radius of NWs, the type of conductivity can be tuned from intrinsic to *p*-type and to metallic.

**Figure 7:**
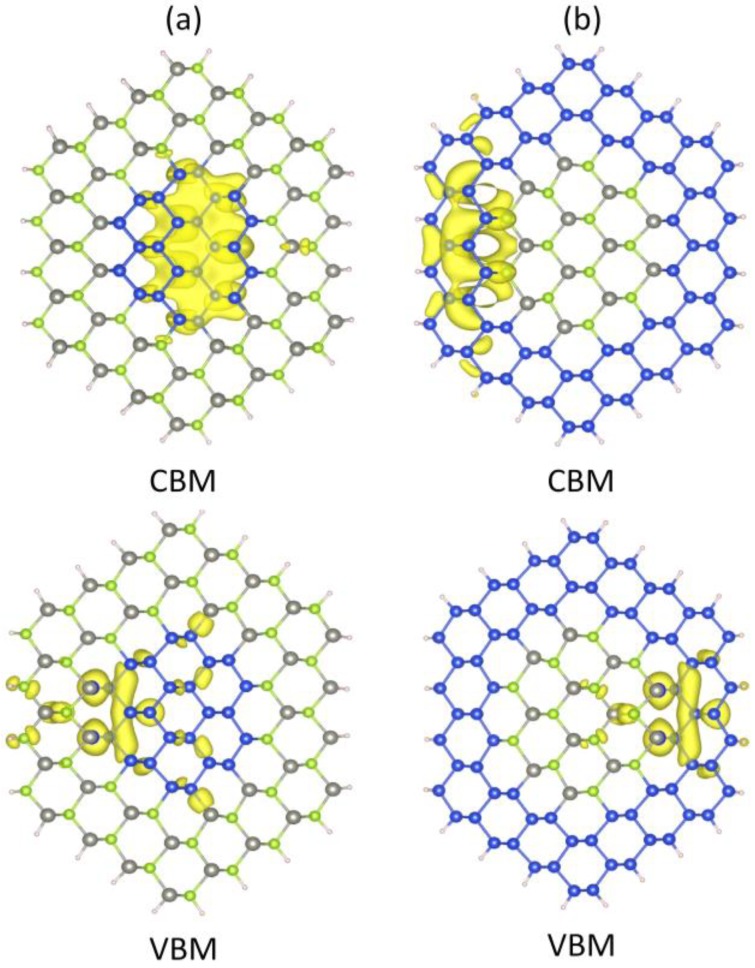
Partial charge density distribution of the CBM and VBM states of (**a**) Si-core/ZnSe-shell and (**b**) ZnSe-core/Si-shell NWs.

Figure 7 depicts the typical charge densities of the CBM and VBM states of the ZnSe-core/Si-shell and Si-core/ZnSe-shell NWs. For Si-core/ZnSe-shell NWs, the CBM is mainly located at the inner Si-core, while the atoms near the interface contribute most of the VBM. As for ZnSe-core/Si-shell NWs, the contribution to the CBM mainly stems from the outer Si shell and ZnSe atoms on the interface. However, the VBM also comes from atoms near the interface. This result indicates that electrons would be confined in the Si part for both core-shell NWs due to the band alignment, while holes are restricted in the interface. According to the type I heterostructure theory, both CBM and VBM would be confined in the Si part. The contradiction in the VBM leads us to believe that the VBM in the ZnSe-core/Si-shell and Si-core/ZnSe-shell NWs is formed by surface states caused by lattice mismatch. 

**Figure 8:**
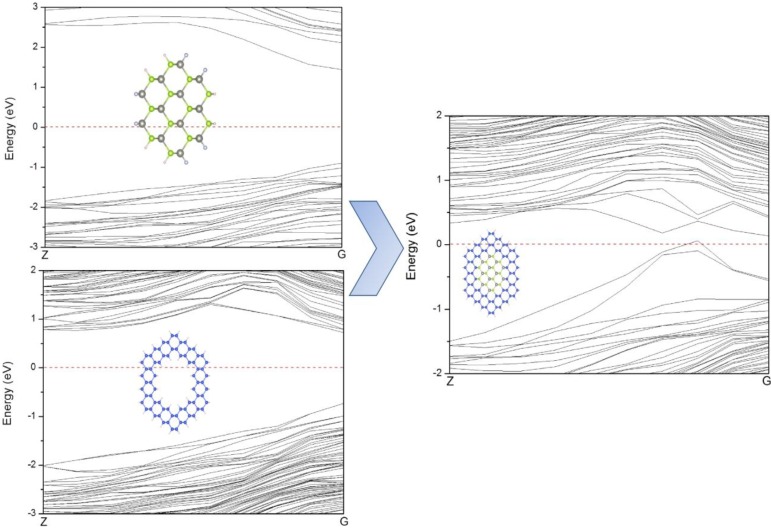
The band structures of ZnSe NWs W1, NTs N2 and ZnSe-core (2.5, 0.25) NWs, which can be considered to be composed of W1 and N2.

Figure 8 illustrates the band structures of ZnSe-core, Si-shell and ZnSe-core/Si-shell NWs. It can be seen that there is no resemblance of VBM between Si-shell and ZnSe-core/Si-shell NWs, indicating that the VBM of ZnSe-core/Si-shell NWs is dominated by interface states. To further illustrate this we depict the schematic of the band alignment of ZnSe-core/Si-shell NWs, where the band edge diagrams before forming the heterostructure are taken from ZnSe NWs and Si NTs.

**Figure 9:**
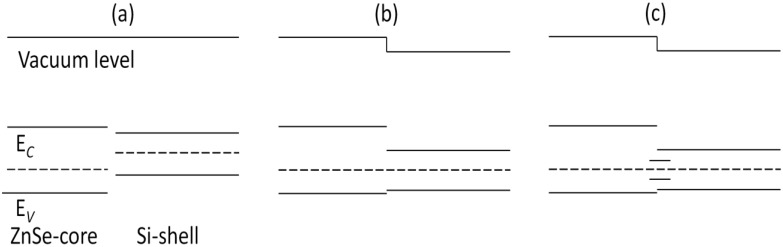
The band alignment of ZnSe-core/Si-shell NWs (**a**) before and (**b**) after the formation of heterostructures; (**c**) the band alignment of NWs with interface states.

**Figure 10:**
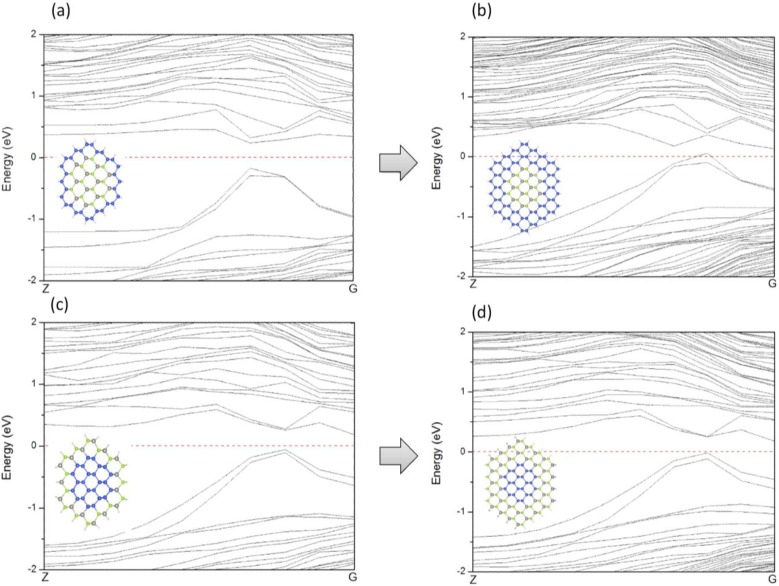
Variation of the band structures of ZnSe-core/Si-shell NWs (**a**,**b**) and Si-core/ZnSe-shell NWs (**c**,**d**). (**a**) ZnSe-core (2.0, 0.44); (**b**) ZnSe-core (2.8, 0.25); (**c**) Si-core (2.0, 0.44) and (**d**) Si-core (2.8, 0.25).

Figure 9a shows the band edge alignment of typical ZnSe-core/Si-shell NWs before forming heterostructures. The ZnSe-core is calculated from ZnSe NWs with a smaller diameter, while the Si-shell part is calculated from Si NTs. Figure 9b is the energy-band profile at equilibrium when a junction is formed, if no interface states exist. Due to the large quantum size effects, the band gaps of both the ZnSe-core and Si-shell part have enlarged. In this example, the Fermi level of the Si-shell is located above that of the ZnSe-core, and they form a type I heterojunction. As can be seen in Figure 9b, the CBM of the Si-shell has a relatively lower energy, and most electrons would be confined in the Si shell. The VBM of the Si-shell is higher than that of the ZnSe-core; however, the valence band alignment is much smaller than the conduction band alignment, Δ*E_V_* < Δ*E_C_*, and thus, the holes tend not to be confined in the Si-shell. As the lattice mismatch between ZnSe and Si is 5.2% and the interface area is large compared to the cell volume, the density of the interface state can be very high; thus, the interface states would be introduced in the band gap. These newly introduced interface states would be bent in the reciprocal space and form a new VBM, and the band gap is reduced correspondingly. This result is consistent with what is shown in Figure 6 and the partial charge density distribution of CBM and VBM states. By using the band edge alignment, we can also explain why the conductivity type of ZnSe-core/Si-shell NWs can be tuned by shell thickness, but that of Si-core/ZnSe-shell NWs cannot. As presented in Figure 10, both ZnSe-core/Si-shell and Si-core/ZnSe-shell NWs have fixed core diameters and varying shell thicknesses; thus, the amount of surface states can be deemed as the same, and the shape of the VBM remains unchanged when the shell swells. However, for ZnSe-core/Si-shell NWs, a thicker Si shell would reduce the band gap of NWs because a thicker Si shell would have smaller band gaps. This is what is shown in Figure 10a,b. A further increase of the shell thickness would make the NWs metallic (the result is not shown here). For Si-core/ZnSe-shell NWs, a thicker ZnSe shell would not affect the band gap and conductivity type of NWs, since the band gap of the Si core is still smaller than that of the ZnSe shell, and the band structure is mainly determined by Si and the interface states, as is exactly seen in Figure 10c,d.

## 4. Conclusions

In conclusion, we have investigated the electronic properties of ZnSe/Si core-shell NWs along the [110] direction by employing first principle calculation. To understand the role of the core and shell part, we built ZnSe NT structures and find that NTs have larger band gaps than NWs of the same diameter, due to the smaller cross-section areas that they have. Additionally, the band gap decreases with the increasing of the thickness of the NT. The VBMs of ZnSe NT are degenerate, while those of ZnSe NWs are not. The VBM states of both NTs and NWs come from the 4*p* state of Se atoms, and the CBM state is a joint contribution of the *s* orbital of Se and the *sp^3^* orbital, consisting of the Se 4*p* and Zn 4*s* orbitals. The band gaps of both ZnSe-core/Si-shell NWs and Si-core/ZnSe-shell NWs are smaller than those of pure ZnSe or Si NWs, and ZnSe-core/Si-shell NWs would turn from intrinsic to *p*-type, then to metallic characteristics when the shell thickness is enlarged. However, Si-core/ZnSe-shell NWs would remain intrinsic by varying the shell thickness. The partial charge distribution diagram shows that CBM would be confined in the Si part, while VBM is mainly distributed in atoms on the interface. By using the band edge alignment at equilibrium of the heterostructures, we conclude that the small band gap is the result of the introduction of interface states into the band gap.
